# Rare incidence of primary adrenocortical carcinosarcoma: A case report and literature review

**DOI:** 10.3892/ol.2014.2635

**Published:** 2014-10-24

**Authors:** YONG-BAO WEI, YUN-LIANG GAO, HONG-TAO WU, SHI-FENG OU-YANG, TAO XU, DONG-FANG MAO, JIN-RUI YANG

**Affiliations:** Department of Urology, The Second Xiangya Hospital, Central South University, Changsha, Hunan 410011, P.R. China

**Keywords:** adrenocortical carcinosarcoma, adrenocortical carcinoma, pathology

## Abstract

Adrenocortical carcinoma (ACC) is a rare, but highly aggressive type of tumor with an incidence of one to two per million annually. Adrenocortical carcinosarcoma is an exceptional variant of ACC, which is characterized by the presence of histological regions of carcinoma and sarcoma. To date, to the best of our knowledge, there have only been 12 reported cases of adrenocortical carcinosarcoma. In the present study, a case of primary, non-functional adrenocortical carcinosarcoma is described, as well as a review of the literature to raise awareness of this particularly rare type of malignant neoplasm that is associated with a worse diagnosis and prognosis than adrenocortical carcinoma. In the present study, the patient underwent a laparoscopic left adrenalectomy and the tumor was dissected without complication from the left kidney. Microscopic observations showed the tumor comprised of epithelial and spindle cell components. The patient did not exhibit signs of tumor recurrence at the one-month follow-up. The potential diagnosis of adrenocortical carcinosarcoma must be considered when diagnosing adrenal malignancies in adults. In addition, comphrensive imunohistochemical staining may be required to identify possible sarcomatous patterns. To the best of our knowledge, the present case is the first to report an incidence of adrenocortical carcinosarcoma in China. Details of the patient are presented and the pathology of adrenocortical carcinosarcoma is discussed.

## Introduction

Adrenocortical carcinoma (ACC) is a rare, but highly aggressive type of tumor with an incidence of one to two per million annually (1–3). Furthermore, a higher incidence of ACC has been reported in females. ACC is associated with a bimodal age distribution that has two peaks; one representing children in the first decade of life and the other representing adults in the fourth to fifth decades of life (4–5). A small number of cases of ACC have been reported to date, each with a poor prognosis and a five-year overall survival rate of ~35% (4,6–8). The poor prognosis may be attributable in part to the advanced stage at which the majority of ACCs are detected (4).

Adrenocortical carcinosarcoma is an exceptional variant of ACC, which is characterized by the presence of histological regions of carcinoma and sarcoma, with the latter regularly demonstrating heterologous features, including rhabdomyoblastic (9–12), chondroid or osteogenic differentiation (9,13). The term sarcomatoid carcinoma has also been synonymously used to refer to adrenocortical carcinosarcoma. In the present study, a case of primary, non-functional adrenocortical carcinosarcoma is described and a review of the literature is presented to raise awareness of this particularly rare type of malignant neoplasm with a poor diagnosis and prognosis. To the best of our knowledge, the present case is the first to report an incidence of adrenocortical carcinosarcoma in China. Written informed consent was obtained from the family of the patient.

## Case report

### Patient information

**Figure 1:**
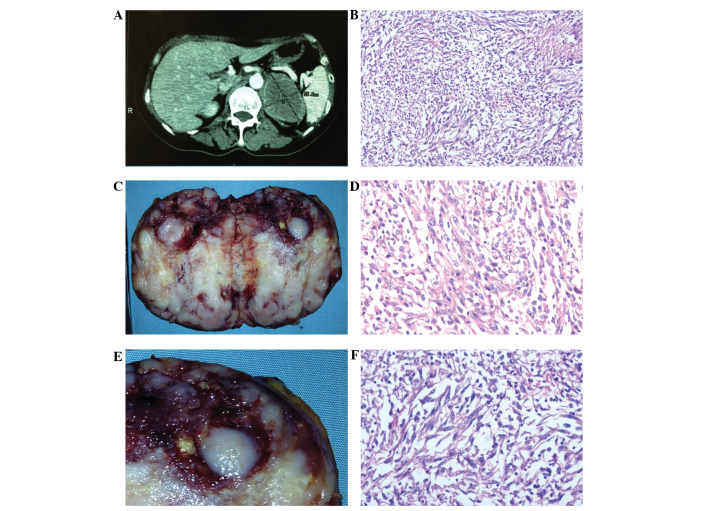
Gross images and hematoxylin and eosin (H&E)-stained microscopic images of the primary adrenocortical carcinosarcoma. (A) A computed tomography scan demonstrates a 7.6×5.1-cm well-demarcated and peripherally enhanced left adrenal mass impinging on the pancreas and the spleen, without parenchymal invasion into the kidney. (B) Gross image of the cut surface of the adrenocortical carcinosarcoma shows a circumscribed mass, predominantly comprising paler, firm tissue, with partially yellow tissue. (C) A compressed rim of normal adrenal gland is apparent adjacent to the tumor capsule. (D) The tumor comprises of spindle cell and epithelial components (H&E staining; magnification, ×100). (E) Carcinomatous cells exhibit highly atypical nuclei and an abundant eosinophilic cytoplasm (H&E staining; magnification, ×200). (F) Spindle-shaped tumor cells reveal highly pleomorphic nuclei (H&E staining; magnification, ×200).

In November 2013, a 63-year-old female was admitted to the Second Xiangya Hospital (Changsha, China) with left flank pain, fatigue, decreased appetite and weight loss that had persisted for three months. These clinical symptoms led to the discovery of a 7-cm heterogeneous hypoechoic left adrenal mass on an abdominal ultrasound. On admission to the Department of Urology at the Second Xiangya Hospital, the results of the physical examination were normal and there was no palpable abdominal mass. The patient exhibited no clinical features associated with excessive steroid hormone or catecholamine levels and had no notable family medical history. Furthermore, laboratory studies of the adrenal hormone levels of cortisol, aldosterone and catecholamines were within the normal limits. 24-h urine volume was 1100 ml (normal range, 1000–1500 ml), 24-h urinary vanillylmandelic acid level was 33.1 μmol/day (normal range, 0–68.6 μmol/day), the clinostatic and orthostatic plasma renin activity levels were 270 ng/l/h (normal range, 150–2330 ng/l/h) and 388 ng/l/h (normal range, 100–6560 ng/l/h), respectively. The clinostatic and orthostatic plasma aldosterone levels were 56 ng/l (normal range, 30–160 ng/l) and 119 ng/l (normal range, 70–300 ng/l), respectively. A dynamic, contrast-enhanced abdominal computed tomography scan showed a 7.6×5.1-cm well-demarcated and peripherally enhanced left adrenal mass that was impinging on the pancreas and the spleen, without parenchymal invasion into the kidney (Fig. 1A). Distant lymph node, pulmonary or liver metastases were not observed. A laparoscopic left adrenalectomy was performed and the tumor was dissected without complication from the left kidney. The patient did not exhibit signs of tumor recurrence at the one-month follow-up.

### Gross pathology

The gross specimen presented as a well-demarcated, encapsulated spherical mass, which measured 8×6×4 cm. The cut surface was firm and varied in color. The majority of the lesion was pale, however, certain regions (~30% of the tumor area) were bright yellow (Fig. 1B). Approximately 20% of the tumor area comprised of numerous foci of necrosis. There was a small quantity of the residual adrenal gland, which appeared to be laminated at the periphery of the tumor (Fig. 1C). The spleen, pancreas and kidney had not been invaded by the tumor.

### Microscopic observations

The tumor comprised of epithelial and spindle cell components that were multifocally intermingled (Fig. 1D and E). In addition, focal necrosis and hemorrhaging was observed. The main epithelial component exhibited poorly differentiated carcinomatous cells, which were arranged in a diffuse and solid growth pattern. These cells showed highly atypical nuclei, and an abundant eosinophilic cytoplasm and usually possessed large, prominent nucleoli (Fig. 1E). Furthermore, spindle-shaped tumor cells were observed to be arranged in a haphazard fascicular pattern with highly pleomorphic nuclei (Fig. 1F).

Variable numbers of cells undergoing mitosis were identified in different areas. The highest mitotic activity was observed in the sarcomatous area, however, only occasional instances of mitosis were observed in the carcinomatous area. Furthermore, no definite capsular invasion was observed histologically.

### Immunohistochemical findings

**Figure 2:**
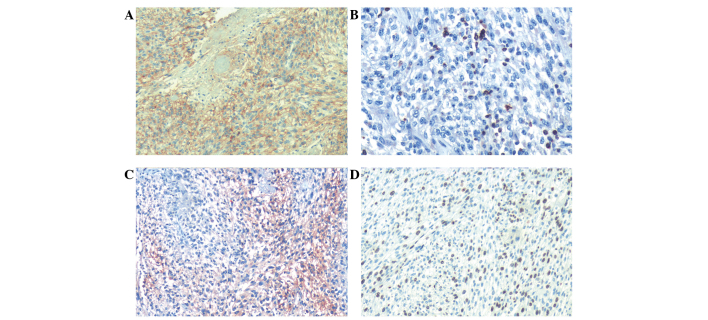
Immunohistochemical staining of the primary adrenocortical carcinosarcoma. (A) Carcinomatous and sarcomatous cells positive for cluster of differentiation 56 (magnification, ×200). Cytoplasm of the sarcomatous cells positive for (B) Bcl-2 (magnification, ×200) and (C) neuron specific enolase (magnification, ×100). (D) Carcinomatous and sarcomatous cells positive for Ki-67 (x100).

An extensive immunohistochemical panel was performed on formalin-fixed, paraffin-embedded tumor tissue to evaluate the sarcomatous and carcinomatous components. In addition, negative and positive controls were performed for each immunohistochemical stain, including cytokeratin (CK), synapsin (Syn), CD56, CD34, neuron-specific enolase (NSE), Ki-67, S100, smooth muscle actin, Bcl-2, human melanoma black 45 (HMB-45) and chromogranin A (CgA). Negative controls included primary antigens (rabbit anti-human antibodies), while positive control included the aforementioned primary antigens and the secondary (goat anti-rabbit) antibodies. Cluster of differentiation (CD)56 demonstrated diffuse positivity in the epithelial cells and spindle areas (Fig. 2A). Bcl-2 showed focal positivity in the sarcomatous areas (Fig. 2B) and CD34 was weakly positive. NSE was only weakly positive in sarcomatous components (Fig. 2C) and Ki-67 demonstrated 70% positivity in the two components (Fig. 2D). S-100 showed equal amounts of positivity/negativity, and CK, Syn, smooth muscle actin, HMB-45 and CgA showed no reactivity in all of the areas.

## Discussion

ACCs containing a component of sarcoma or sarcoma-like (spindle cells) differentiation are a particularly rare and aggressive type of neoplasm, and, to the best of our knowledge, only 12 cases have previously been reported in the English literature to date (9–20). The clinical and pathological features of reported patients presenting with this type of tumor, including the present case, are summarized in Table I (9–20). The age of initial presentation ranged from 23 to 79 years, with a mean of 50.3 years and a median of 45 years, which appears to be similar to the results of a previous study (mean age, 40–50 years) by Sasaki *et al* (11). Eight of the 13 patients were aged <50 years. Compared with previous studies of ACC (4), adrenocortical carcinosarcoma also showed a female preponderance with a female/male ratio of 1.6:1. Flank/abdominal pain or discomfort was identified to be a common presentation (10/13 cases) and the majority of adrenocortical carcinosarcoma presented in the left adrenal gland (9/12 cases). One tumor was identified during an investigation of a rectal mass in a pregnant patient (19). In the majority of cases, these tumors did not exhibit any endocrine dysfunction, although three of the 13 cases were associated with corticosteroid hypersecretion (10,13,16). Generally, the tumors were particularly large (mean size, 14.1 cm; weight, 1,743 g) and exhibited dramatically aggressive behavior. All 13 patients succumbed to their cancer within the range of two days to 14 months following resection, despite aggressive administration of multimodality therapeutic strategies.

The present case was extensively sampled, however, the heterologous elements, such as rhabdomyosarcoma (9) and osteosarcoma (13), that were documented in certain previous papers were not observed. Additionally, when compared with previous studies, in this study there were no specific immunohistochemical staining results. The positive reactivity with NSE that was only observed in partial sarcomatous areas may provide evidence for neuroendocrine differentiation in ACCs, which is consistent with two previous studies; one demonstrated positive staining for NSE, Syn and neurofilament protein (21), and the other showed positivity for CK AE1/AE3, Syn and NSE (20).

Notably, the tumor mass observed in the present case, (size, 8×6×4 cm) was the smallest out of all of the reported cases. The next smallest, measuring 9×7.5×6.5 cm, was reported in 1993 (13). The tumor sizes described in the other 11 cases were all >10 cm, which indicates that although a tumor mass may not be large it may be an adrenocortical carcinosarcoma. Therefore, the diagnosis of adrenocortical carcinosarcoma may be complicated.

Furthermore, a compressed rim of normal adrenal gland was observed adjacent to the tumor capsule in the present study, which was also described in two previous cases (12,20) and in a case of large diameter ACC (22). Therefore it may be hypothesized that the tumor arises from an accessory/ectopic adrenal gland, or alternatively, from a nodular area of the adrenal gland, which gradually becomes entirely replaced by the tumor (22).

A challenge during the diagnosis of adrenocortical carcinosarcoma arises from the difficulty in grossly identifying the tumor origin. Renal carcinosarcoma, metastatic melanoma and primary retroperitoneal sarcoma should be considered in the differential diagnosis, and attention should be given to ACCs demonstrating negative, or only focally weak, positivity for CKs. Consequently, it is necessary to adopt immunohistochemical staining for melan-A, inhibin and calretinin to verify the adrenocortical origin, particularly for ACC (23–26). Routine histological data from Table II revealed that the carcinomatous and sarcomatous components expressed vimentin. A notable feature is that desmin was found to be highly expressed in the sarcomatous component. Furthermore, the sarcomatous component of the tumor was positive for HHF, myogenin, and caldesmon, whereas the epithelial component was diffusely positive for inhibin, melan-A, S-100 protein, NSE and HMB-45. As a result, a large number of tumor samples and an extensive panel of immunohistochemical staining are considered to be necessary to identify the adrenal origin and the biphasic components of adrenocortical carcinosarcoma.

In conclusion, a potential diagnosis of adrenocortical carcinosarcoma should be considered when diagnosing an adrenal malignancy in adult patients. Extensive sampling of the tumor together with comprehensive immunohistochemical staining are required to identify a possible sarcomatous pattern and, as a result of the poor prognosis associated with adrenocortical carcinosarcoma, the most aggressive therapeutic regimens are required.

## Figures and Tables

**Table I:** Clinical and pathological features of previously reported cases of adrenocortical carcinosarcoma.

Ref.	Author, year	Age, years/gender	Clinical presentation	Endocrine dysfunction	Location	Size/weight	Sarcomatous component	Immunostaining results		Total number of antibodies	POS

								Caricinomatous	Sarcomatous		
14	Okazumi *et al,* 1987	46/M	Abdominal distention	No	R	14 cm, 880 g	Spindle cell	NA	NA	NA	6 months
15	Collina *et al,* 1989	68/F	Abdominal discomfort	No	L	11 cm NA	Spindle cell	Low molecular weight CK, Vim	Low molecular weight CK, Vim	7	6 months
9	Decorato *et al,* 1990	42/F	Abdominal pain	No	L	19 cm, 1400 g	Rhabdo-myosarcoma	-	muscle specific actin	5	7 months
10	Fischler *et al,* 1992	29/F	↓ Weight, virilization	Yes	L	12.5 cm, 610 g	Rhabdo-myosarcoma	Vim,	Vim, HHF and desmin	5	8 months
13	Barksdale *et al,* 1993	79/F	Hypertension	Yes	R	9 cm, 199 g	Osteosarcoma, chondrosarcoma	Vim	Vim	5	NA
16	Lee *et al,* 1997	61/M	Flank pain	Yes	R	12 cm NA	Spindle cell	CK, focal NSE	CK, Vim	10	2 days
17	Sturm *et al,* 2008	31/M	Abdominal pain	No	L	12 cm, 620 g	Spindle cell	CD56, Vim, focal desmin, CK AE1/AE3, α-inhibin, Syn	CD56, Vim, focal desmin, HHF35	17	3 months
18	Coli *et al,* 2010	75/F	Abdominal pain	No	L	15 cm NA	Spindle cell	CK (MNF-16), Vim, S-100, melan-A, focal HMB-45	Vim, SMA, desmin, caldesmon, myogenin	20	12 months
11	Sasaki *et al,* 2010	45/M	Abdominal pain	No	L	17 cm, 2974 g	Rhabdo-myosarcoma	Vim, Syn, melan-A, and calretinin	Vim, Syn, melan-A, and calretinin; desmin, myogenin, myoglobin	15	3 months
19	Bertolini *et al,* 2011	23/F	Fatigue, ↓ appetite, fixed mass in rectum	No	L	14 cm NA	Osteosarcomatous	Inhibin, calretinin	-	NA	14 months
12	Thway *et al,* 2012	45/M	Bloating, back pain	No	L	24 cm, 6500 g	Rhabdo-myosarcoma	Melan-A, CD56, MNF116	Desmin, myogenin	28	11 months
20	Kao *et al*, 2013	45/M	Abdominal pain, weight loss	No	R	15 cm, 760 g	Spindle cells, undifferentiated	melan-A, inhibin, calretinin, AE1/AE3, Syn, Vim,	Vim, NSE, FLI-1; Undifferentiated: CD99	18	7 months ; alive
Current study	Wei *et al*	63/F	Fatigue, flank pain	No	L	8 cm NA	Spindle cells	NSE, FLI-1 CD56, Ki-67	CD56, Bcl-2, NSE, Ki-67	11	1 month ; alive

M, Male; F, Female; R, right; L, left; NA, not available; CK, cytokeratin; Vim, vimentin; -, negative, no carcinomatous or sarcomatous regions; NSE, neuron specific enolase; CD, cluster of differentiation; Syn, synaptophysin; POS, postoperative survival.

**Table II:** Immunohistochemical results (not including cytokeratins) of the reported adrenocortical carcinosarcoma.

A, Carcinomatous component			

Antibody	Check sum	Positive	Positive rate
FLI-1	1	1	100
Melan-A	5	4	80
Vimentin	9	7	78
CD56	4	3	75
Inhibin	6	3	50
Ki-67	2	1	50
Syn	7	3	43
NSE	5	2	40
HMB-45	5	1	20
Desmin	7	1	14
S-100	8	1	13

B, Sarcomatous component			

Antibody	Check sum	Positive	Positive rate

HHF	3	3	100
Bcl-2	1	1	100
FLI-1	1	1	100
Vimentin	9	8	89
Desmin	7	5	71
CD56	4	2	50
Caldesmon	2	1	50
Ki-67	2	1	50
Myoglobin	2	1	50
Myogenin	5	2	40
NSE	5	2	40
Calretinin	5	1	20
Melan-A	5	1	20
SMA	6	1	17
Syn	7	1	14

CD, cluster of differentiation; Syn, synaptophysin; NSE, neuron specific enolase; HMB-45, human melanoma black 45; SMA, smooth muscle actin.
